# Population-based screening for conditions associated with juvenile sudden cardiac death: a systematic review and meta-analysis

**DOI:** 10.1093/ehjqcco/qcag004

**Published:** 2026-01-28

**Authors:** Francesca Bonanni, Angelo Capodici, Francesco Gentile, Francesca Moschetti, Claudio Passino, Marco Di Paolo, Iacopo Olivotto, Michele Emdin, Alberto Giannoni

**Affiliations:** Health Science Interdisciplinary Centre, Scuola Superiore Sant’Anna, Piazza Martiri della Libertà 33, 56127 Pisa, Italy; Health Science Interdisciplinary Centre, Scuola Superiore Sant’Anna, Piazza Martiri della Libertà 33, 56127 Pisa, Italy; Health Science Interdisciplinary Centre, Scuola Superiore Sant’Anna, Piazza Martiri della Libertà 33, 56127 Pisa, Italy; Cardiovascular Medicine Division, Fondazione Toscana G. Monasterio, via Giuseppe Moruzzi 1, 56124 Pisa, Italy; Health Science Interdisciplinary Centre, Scuola Superiore Sant’Anna, Piazza Martiri della Libertà 33, 56127 Pisa, Italy; Health Science Interdisciplinary Centre, Scuola Superiore Sant’Anna, Piazza Martiri della Libertà 33, 56127 Pisa, Italy; Cardiovascular Medicine Division, Fondazione Toscana G. Monasterio, via Giuseppe Moruzzi 1, 56124 Pisa, Italy; Department of Surgical Pathology, Medical, Molecular and Critical Area, Institute of Legal Medicine, University of Pisa, Lungarno Pacinotti 43, 56126 Pisa, Italy; Department of Experimental and Clinical Medicine, University of Florence, Largo Brambilla 3, 50134 Florence, Italy; Meyer Children’s Hospital IRCCS, Viale Gaetano Pieraccini 24, 50139 Florence, Italy; Health Science Interdisciplinary Centre, Scuola Superiore Sant’Anna, Piazza Martiri della Libertà 33, 56127 Pisa, Italy; Cardiovascular Medicine Division, Fondazione Toscana G. Monasterio, via Giuseppe Moruzzi 1, 56124 Pisa, Italy; Health Science Interdisciplinary Centre, Scuola Superiore Sant’Anna, Piazza Martiri della Libertà 33, 56127 Pisa, Italy; Cardiovascular Medicine Division, Fondazione Toscana G. Monasterio, via Giuseppe Moruzzi 1, 56124 Pisa, Italy

**Keywords:** Sudden cardiac death, Cardiovascular screening, Inherited and congenital heart diseases, Children, Adolescents, Electrocardiography, Primary prevention, Public health, Mass screening, Early diagnosis

## Abstract

**Aims:**

Juvenile sudden cardiac death (SCD) is predominantly caused by inherited or congenital heart conditions. While structured screening programmes for competitive athletes are well established, current international guidelines emphasize a gap of evidence regarding the value of mass screening in the general youth population. This systematic review aimed to assess the effectiveness of large-scale screening programmes in detecting conditions associated with SCD among young individuals.

**Methods and results:**

We conducted a systematic review following PRISMA guidelines, with the protocol registered in PROSPERO (CRD42024540606). Original studies evaluating cardiovascular screening in young individuals were included, while studies exclusively involving competitive athletes were excluded. Nineteen studies encompassing 1 079 781 participants from multiple countries were finally analysed. Most studies (68%) were published recently and primarily targeted children and adolescents aged 6–19 years; 63% were assessed as having a low risk of bias. Screening modalities included electrocardiography (ECG) alone (26.3%), questionnaires alone (5.3%), and a combination of both (68.4%). Following second- or third-line investigations, the overall diagnostic yield for SCD-risk conditions was 1.4‰ (1.9‰ in the meta-analysis). The pooled prevalence, estimated using a random-effects model, was 2.23‰ (95% CI: 0.94–5.29‰), with extremely high heterogeneity among studies (*I*^2^ = 100%, *P* < 0.0001).

**Conclusion:**

Systematic cardiovascular screening may help identify young individuals at increased risk for SCD, providing a potentially meaningful diagnostic yield. However, the absence of long-term data on outcome and cost-effectiveness underscores the need for further research to refine screening protocols, assess sustainability, and inform evidence-based public health policies.

## Graphical Abstract

**Graphical Abstract:**
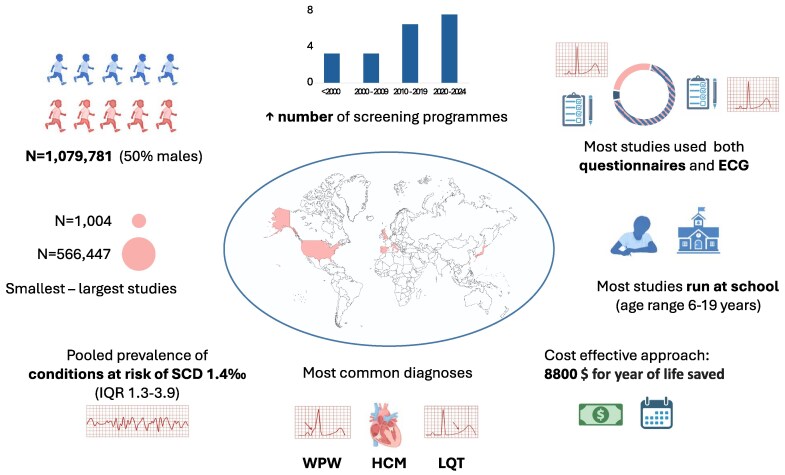

Key Learning Points **What is already known:**

- Juvenile sudden cardiac death (SCD) is most often caused by previously undiagnosed inherited or congenital heart conditions.
- The majority of SCD events in young individuals occur outside competitive sports.
- While pre-participation screening is established for competitive athletes, current international guidelines do not recommend mass screening for the general youth population due to insufficient evidence of benefit.

**What this study adds:**

- This systematic review and meta-analysis including over one million individuals shows that large-scale screening identifies SCD-risk conditions in ∼1.4‰ young people (1.89‰ in the meta-analysis), most commonly Wolff-Parkinson-White syndrome, long QT syndrome, and hypertrophic cardiomyopathy.
- The diagnostic yield and screening approaches vary significantly across studies (*I*^2^ ≈ 100%), highlighting the urgent need for standardized protocols and reporting criteria.
- There remains a critical lack of data on long-term outcomes and cost-effectiveness, which is essential to guide evidence-based public health policies regarding cardiovascular screening in the young.

## Introduction

Sudden cardiac death (SCD) is a major public health issue,^1^ especially in young individuals, due to the profound emotional and social repercussions of a premature loss of life.

While coronary artery disease is predominant in subjects over 50 years of age,^2^ juvenile SCD is largely due to inherited conditions,^3^ including cardiomyopathies, such as hypertrophic cardiomyopathy (HCM), arrhythmogenic cardiomyopathy (ACM), dilated cardiomyopathy, or channelopathies, such as long QT syndrome (LQTS), short QT syndrome (SQTS), Brugada syndrome (BRS), and catecholaminergic polymorphic ventricular tachycardia (CPVT).^4^ Other causes are coronary artery anomalies (CAAs),^5^ connective tissue disorders,^6^ Wolff-Parkinson-White syndrome (WPWS),^7^ myocarditis,^8^ and some congenital heart diseases (CHD).^9^

Beyond the myocardial substrate, specific triggers contribute to arrhythmic events. Extreme physical exertion in athletes has long been considered a major risk factor in susceptible individuals.^9^ The Veneto region in Italy pioneered pre-participation cardiovascular screening in athletes to identify such a risk.^10^ Accordingly, ACC/AHA^11^ and ESC guidelines^12^ have recognized the risk of SCD in athletes and the latter has also recommended cardiovascular evaluation, including history, physical exam, and ECG (Class IIA, Level C).

While illicit substances and energy drinks have been implicated as potential contributing factors in some cases of juvenile SCD,^13,14^ the overall evidence remains limited, apart from a recent large longitudinal study that excluded adolescents (<18 years), but demonstrated a higher risk of ventricular arrhythmias and cardiac arrest, especially in younger adults using methamphetamine or cocaine.^15^ Nonetheless, most events appear to occur during rest or recreational activities rather than competitive sports,^16,17^ with forensic data consistently showing a higher incidence of SCD among non-athletes than among competitive athletes.^16^

Recent analyses have documented marked variability in cardiac screening practices across diverse countries and target populations.^18,19^ A key distinction emerges between well-established protocols for competitive athletes and the less-defined strategies for the general population. While pre-participation screening for athletes has become progressively standardized and often guided by consensus frameworks,^11,12^ the evidence supporting the effectiveness of mass screening in the general young population remains limited and controversial,^20^ largely dependent on national policies, local healthcare resources, and historical practice.^21^ Notably, the debate surrounding population-level screening does not reflect a lack of clinical relevance, but rather a paucity of high-quality, comparative data in non-athletic cohorts.^12^

This systematic review and meta-analysis was designed to address this knowledge gap by consolidating evidence from real-world screening programmes targeting the general young population. By evaluating programme design, diagnostic yield, downstream investigations, and clinical outcomes, this work aims to provide a comprehensive, data-driven overview to inform the development of rational, proportionate, and context-specific screening strategies.

## Methods

A systematic review was conducted following the Preferred Reporting Items for Systematic Reviews (PRISMA) approach.^22^ The protocol was registered on PROSPERO (CRD42024540606). Analysing pre-existing data, patient and public involvement was deemed not appropriate. Data from this review will be made available from the corresponding author upon request.

### Search strategy

The search query launched on 25 April 2024, included peer-reviewed publications on PubMed and Scopus using the following search string:

- PubMed: (screeni* OR detect* OR prevalence) AND (sudden cardiac death OR SADS) AND (juveni* OR adolesce* OR youth OR young OR school OR child*) NOT Review.
- Scopus: TITLE-ABS-KEY (screeni* OR detect* OR prevalence) AND TITLE-ABS-KEY (sudden AND cardiac AND death OR sads) AND TITLE-ABS-KEY (juveni* OR adolesce* OR youth OR young OR school OR child*) AND (EXCLUDE (DOCTYPE, ‘re’)).

For the purposes of this review, we adopted a broader inclusion criterion for "young" people, including participants up to 45 years of age, to capture all large-scale population cardiovascular screening studies that included significant cohorts of adolescents and young adults, thereby providing a comprehensive overview of the existing evidence. This threshold was also chosen to include all studies focusing on populations for whom preventive strategies for SCD, such as time-dependent care networks for myocardial infarction and stroke, are not yet established.

Studies exclusively targeting competitive athletes were excluded, as pre-participation screening in this group is already widely implemented and extensively studied. However, studies including both athletes and non-athletes were retained to capture data representative of the general young population and real-world screening practices. A competitive athlete was defined as an individual who engages in regular training and participates in organized sports competitions to achieve high performance.

### Data collection

Two reviewers (FB and AC) independently conducted title screening, abstract screening and data extraction. Any disagreement was resolved through discussion.

Variables extracted included: population age (range in years), population sex (% males), race and ethnicity, total number of participants, exclusion of pre-existing cardiac diagnoses (yes/no), type of screening conducted (questionnaires and/or electrocardiograms—ECG), ECG performed by clinicians (yes/no), number of subjects with family history for juvenile SCD or SCD-risk conditions, number of subjects with cardiological symptoms/signs, number of ECG alterations identified, number of subjects undergoing second- or third-line investigations, total number of cardiological diagnoses made, total number of cardiological diagnoses associated with a higher risk of juvenile SCD, number of patients who experienced SCD.

All cardiomyopathies, channelopathies, WPW syndrome, previous myocarditis, and the presence of ventricular tachycardia (VT), ventricular fibrillation (VF), or third-degree atrioventricular block (AVB III) were considered diagnoses at risk of juvenile SCD. Selected forms of CHD were also included, particularly those with residual haemodynamic abnormalities, arrhythmic substrates, or cyanotic physiology, which are known to confer an increased risk of SCD.^23^

### Bias assessment

Per registered protocol, the scales used were the ROBINS-I tool for interventional cohort studies and the AXIS tool for the cross-sectional ones. The assessment was performed by two reviewers independently (F.B. and A.C.). Disagreement was resolved through discussion.

### Data analysis

Data were summarized as absolute frequencies and percentages. Prevalence estimates were reported as means with standard deviations (SD) and medians with interquartile ranges (IQR). A pooled mean prevalence was also calculated by aggregating the number of detected cases and dividing by the total number of individuals screened across studies. Categorical variables in different subgroups were compared using the χ^2^ or Fisher’s exact tests. For comparisons of continuous, non-normally distributed variables, the Mann–Whitney U test and the Kruskal–Wallis test were used, as appropriate. Statistical significance was defined as a two-tailed *P*-value <0.05. These analyses were performed using SPSS (IBM Corp., Armonk, NY, USA).

A proportion meta-analysis was conducted to pool the prevalence of detected cardiac conditions and cardiac diseases at risk of SCD. A random-effects model based on a random intercept logistic regression was used for the analysis.

Results were calculated as event rates per 1000 participants, with 95% Confidence Intervals (CI). Between-study heterogeneity was quantified using the *I*^2^ statistic and assessed with Cochran’s Q test. Subgroup analyses were performed by stratifying studies based on screening type and risk of bias assessment.

To assess the robustness of the results, two sensitivity analyses were conducted: a leave-one-out analysis to evaluate the influence of each study on the pooled result, and a secondary analysis excluding studies that reported an event rate higher than 7 per 1000 participants, an arbitrary threshold to exclude outlier studies. The risk of publication bias was visually assessed using funnel plots. These analyses were performed in R statistical software (4.3).

### Role of the funding source

The funder of the study had no role in study design, data collection, data analysis, data interpretation, or writing of the report.

## Results

**Figure 1:**
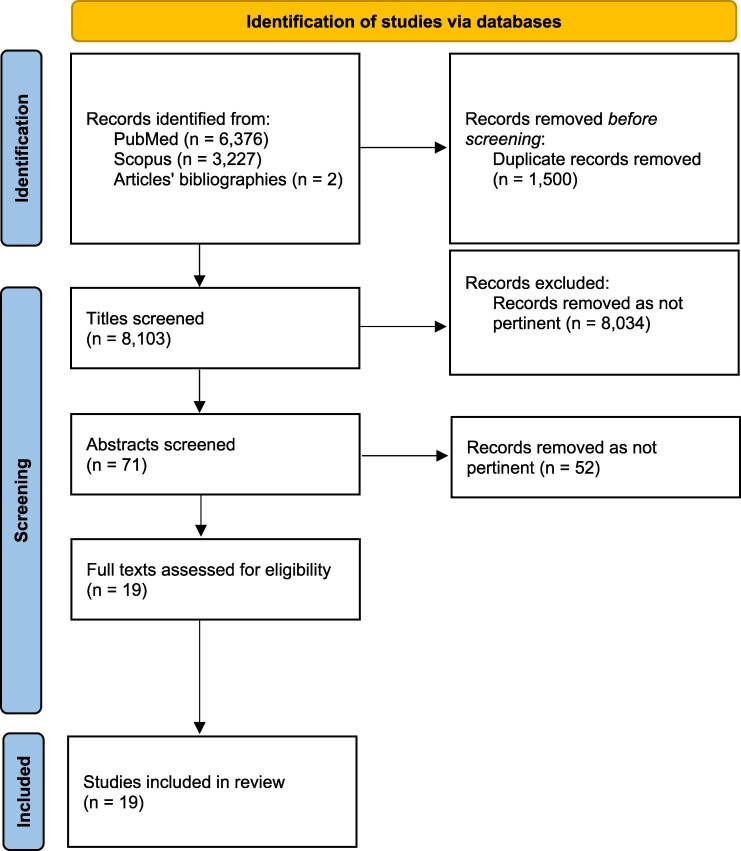
‘Preferred reporting items for systematic review and meta-analysis’ flow chart.

A total of 6376 articles were retrieved from PubMed and 3227 from Scopus. We removed 1500 duplicate records, 8034 articles after title screening, and 52 articles after abstract review. Full-text screening resulted in the selection of 17 articles, with additional 2 articles identified through reference list screening of relevant publications. In total, 19 studies were included in the final analysis, as shown in *Figure 1*.

**Figure 2:**
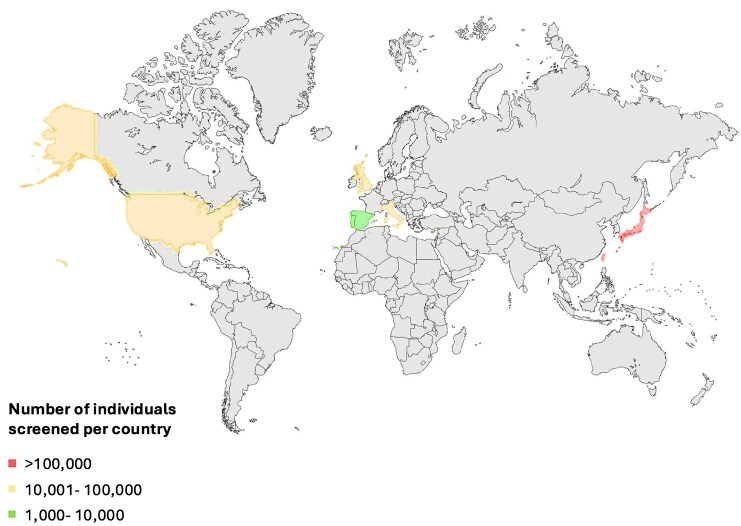
Geographical representation of the different screening campaigns in the world. Studies with a number of individuals screened per country from 1000 to 10,000, from 10 001 to 100 000 and >100 000 are represented in green, yellow, and red, respectively.

The included studies were predominantly conducted in the UK and Japan (4 out of 19 studies each), followed by the USA (3 studies), Italy and Taiwan (2 studies each), Malta, Spain, Lebanon, and Portugal (1 study each). Overall, 88% of the population screened was from Asia, 7% from Europe, and 5% from the USA. Most studies were recent, with 68% of studies published after 2010 and 37% published after 2020. Further details on geographic distribution and publication timeline are presented in *Table 1* and *Figure 2*.

**Table 1:** Main characteristics of the different screening campaigns

First author	Publication Year	Country	Race	Population age (interval years)	Males sex	Number of total participants	Competitive athletes (%)	Type of screening	Family history of heart disease	ECG criteria	Number of clinical alterations	Number of ECG alterations	Number of total participants II level	Follow-up	Bias assessment	Quality Rate Scheme
R.W.A. Jones	1979	UK	Not reported	0	Not reported	1034	··	ECG	··	Not specified	··	129	25	yes	ROBINS-I Low	4
N. Haneda	1986	Japan	Asian	6-15	Not reported	128 454	··	Q + ECG	Number unspecified	Local guidelines	Number unspecified	Number unspecified	Number unspecified	yes	ROBINS-I Low	4
H. Tasaki	1987	Japan	Asian	Not reported	Not reported	39 338	··	Q + ECG	Number unspecified	Not specified	Number unspecified	Number unspecified	2439	yes	ROBINS-I Low	2
K. Niwa	2004	Japan	Asian	5–13	52%	152 322	··	ECG	··	Local Guidelines	··	2765	None	no	AXIS tool Moderate	4
Y. Tanaka	2006	Japan	Asian	12–15	Not reported	68 503	··	Q + ECG	Number unspecified	Not specified	Number unspecified	Number unspecified	1876	yes	ROBINS-I Low	2
M.G. Wilson	2008	UK	Not reported	10–27	Not reported	2720	39.6%	Q + ECG	67^a^	Not specified	67^a^	40	107	no	ROBINS-I Moderate	2
R. Providencia	2010	Portugal	White	22 (mean age)	44%	1472	··	Q	46		403^b^	··	None	no	AXIS tool Serious	4
J. Marek	2011	USA	White (66%)	14–19	51%	32 561	30%	ECG	··	Corrado 2010	··	817	None	no	AXIS tool Low	3
J. Fudge	2014	USA	White (68%)	13–24	49%	1339	80%	Q + ECG	257	Corrado 2010	124^c^	72	586	no	ROBINS-I Low	2
N. Chandra	2014	UK	White (85%)	14–35	74%	11 845	34.5%	Q + ECG	Number unspecified	Corrado 2010	Number unspecified	3038	3038	no	AXIS tool Low	2
C. De Lazzari	2017	Italy	White	16–19	45%	13 016	··	Q + ECG	1770^d^	Corrado 2010	Not recorded	3124	None	no	AXIS tool Low	3
S.R. Nasr	2019	Lebanon	White	Not reported	Not reported	1412	··	ECG	··	Not specified	··	17	Number unspecified	yes	ROBINS-I Serious	4
H.W. Liu	2020	Taiwan	Asian	Not reported	47%	566 447	··	Q + ECG	30 750	Not specified	39129	39427	110431	no	AXIS tool Low	4
P. Vilardell	2020	Spain	Not reported	14 (mean age)	50%	1911	··	ECG	··	Corrado 2010/ Sharma 2018	··	36	36	no	AXIS tool Moderate	4
H. Dhutia	2021	UK	White (90%)	14–35	65%	26 900	··	Q + ECG	408	Corrado 2010	441	2289	2964	yes	ROBINS-I Low	2
A. Austin	2022	USA	White (74%)	12–19	56%	14 846	..	Q + ECG	Number unspecified	Drezner 2013	Number unspecified	Number unspecified	5238	no	AXIS tool Low	3
M. Mancone	2022	Italy	White	13–19	47%	11 949	21%	Q + ECG	Number unspecified	Sharma 2018	3944	1945	Number unspecified	yes	ROBINS-I Low	2
M. Abela	2023	Malta	White (96%)	14–17	50%	2708	38.9%	Q + ECG	10	Drezner 2017	21	99	109	no	ROBINS-I Moderate	4
H.K. Lîm	2023	Taiwan	Asian	12–18	56%	1004	··	Q + ECG	41	National Protocol	107	105	145	no	ROBINS-I Moderate	4

Q, questionnaire; ECG, electrocardiograms.

^a^Serious symptoms and/or family history of SCD.

^b^Syncope on effort, chest pain on effort, and sudden onset palpitation.

^c^Cardiac murmur, Marfan stigmata.

^d^Family history of cardiovascular disease.

Among the eight cross-sectional studies, five^24-28^ had a low risk (63%), two^29,30^ a moderate risk (25%), and one study^31^ a serious risk of bias. For the 11 cohort studies, 7^32-38^ had a low risk (64%), 3^39-41^ a moderate risk (27%), and 1^42^ a serious risk of bias.

### Screening modalities, age, ethnicity, and location of the population screened

As for the screening modality, 13 studies (68.4%)^25-28,32-37,39-41^ used ECG and questionnaires, 5 studies^24,29,30,38,42^ (26.3%) only ECG, and 1 study^31^ (5.3%) only questionnaires. Notably, the latter study only provides a clinical suspicion rather than a final diagnosis.

Questionnaires collected information on family history of heart disease and SCD, personal history of cardiovascular conditions, and symptoms such as syncope, dyspnoea, chest pain, and palpitations. In some cases, additional questions addressed demographics, physical activity, and the use of alcohol, tobacco, and recreational drugs. Some of the questionnaires used were available online.^27,32,33,41^

Among the 18 studies that included ECG analysis, 12 explicitly reported adherence, full or partial, to established interpretation criteria. Most programmes (Abela *et al*., Austin *et al*., Chandra *et al*., De Lazzari *et al*., Dhutia *et al*., Fudge *et al*., Mancone *et al*., Vilardell *et al*., Marek *et al*.) followed international recommendations, including the ESC or International Criteria.^43-45^ Studies conducted in Asia (Niwa *et al*., Lîm *et al*., Haneda *et al*.) applied national or institutional protocols for school-based cardiac screening. A few programmes (Jones *et al*., Liu *et al*., Nasr *et al*., Tanaka *et al*., Tasaki *et al*., Wilson *et al*.) did not specify the interpretation standards adopted.

All studies explicitly included school students, except Jones *et al*.; Providencia *et al*. did not specify whether they were included. Twelve studies^24,25,27,28,32-36,39,40,42^ explicitly stated that the screening was conducted within the school environment. In contrast, four studies^29-31,37^ did not report the screening location, while in three studies^26,38,41^ the screening was not conducted in schools.

Variations in ECG methodologies were noted. Liu *et al*., Tasaki *et al*., and Haneda *et al*. used a 4-lead ECG, Jones *et al*. a 6-lead ECG, and Lîm *et al*. a wireless 12-lead ECG monitor. All other studies used standard 12-lead ECGs. Beyond ECG, Tasaki *et al*. and Haneda *et al*. incorporated radiography into first-level screenings, while Lîm *et al*. used digital auscultation and phonocardiography. Notably, four studies^25,32,40,42^ reported that ECG was performed by non-clinicians. Additionally, Niwa *et al*. used a computer programme for automated ECG evaluation.

A total of 1 079 781 individuals were screened across all studies, with sample sizes ranging from 1004^38^ to 566 447.^28^ The median number of participants was 11 949 (IQR: 1692–35 950). Study characteristics are reported in *Table 1*.

Regarding age, three studies included participants under 10 years old, while 13 studies focused on individuals aged 11–20 years. Four studies examined populations over 20 years old. Two studies provided only the mean age (14 years and 22 years), whereas three did not report the age of the population screened.

Ten studies reported a proportion of males ranging between 40% and 60%, while in two studies it exceeded 60%. Notably, Austin *et al*. reported a lower male participation (15.6%). Six studies did not specify sex distribution.

In terms of ethnicity, 10 (53%) studies predominantly included White participants, ranging from 66% to 93%, while 6 studies primarily involved participants of Asian descent (32%). Three studies did not report ethnicity data.

Athletic status was inconsistently reported, limiting comparisons between athletes and non-athletes. Athlete participation was 21% in Mancone *et al*., 38.9% in Abela *et al*., 30% in Marek *et al*., 34.5% in Chandra *et al*., 80% in Fudge *et al*., 39.6% in Wilson *et al*.

A positive family history was found in 33 349 individuals out of a pooled population of 615 606 (5.4%), based on data from 8 studies. Cardiovascular symptoms or signs were present in 44 349 of 614 539 individuals (7.2%) across 8 studies. Abnormal electrocardiographic (ECG) findings were observed in 53 903 of 827 168 screened participants (6.5%) across 14 studies.

A second or third-level cardiovascular evaluation was explicitly reported by the authors in 12 out of 19 studies (63%), involving 126 994 of 738 595 individuals (17.2%). However, the proportion of individuals undergoing further evaluation varied markedly across studies (range: 1.8–44%, IQR: 3–24%). In 3 studies (16%), the secondary evaluation process was not specified, while in 4 studies (21%), no second- or third-level assessment was conducted.

### Descriptive results: prevalence of cardiac conditions and at risk of juvenile sudden cardiac death

The pooled prevalence of any cardiac condition detected was 15‰ (16 318 cases among 1 079 781 individuals screened). The pooled prevalence of specific cardiac conditions at risk of SCD was 1.4‰ (1505 cases out of 1 079 781 individuals screened). When excluding the largest screening programme (*n* = 566 447), the pooled prevalence of specific SCD-risk conditions was 1.6‰ (823 cases out of 513 334 individuals screened). If we considered only studies with second or third-level evaluations planned, or those with low risk of bias, the prevalence was similar (1.4‰, 1235 out of 880 410, and 1.3‰, 1234 out of 916 232, respectively). Considerable variability in prevalence was observed across studies for all cardiac conditions (range: 0.13–61‰, IQR: 1–11‰) and SCD-risk conditions (range: 0.13–4.3‰, IQR: 1.3–3.9‰).

The prevalence of SCD-risk conditions was similar across different geographical regions (median prevalence: 1.2‰, IQR 0.3–5.4‰ in Asia; 2.0‰, IQR 1.4–4.2‰ in Europe; and 1.9‰, IQR 1.3–2.2‰ in the United States; *P* = 0.55), or using different screening modalities (median: 1.3‰, IQR 1.1–1.5‰ for ECG alone; median: 2.1‰, IQR 1.2–4.9‰ for questionnaires plus ECG; *P* = 0.10). Due to inconsistencies in data reporting, it was not possible to assess whether age and ethnicity influenced the prevalence of high-risk diagnoses.

**Figure 3:**
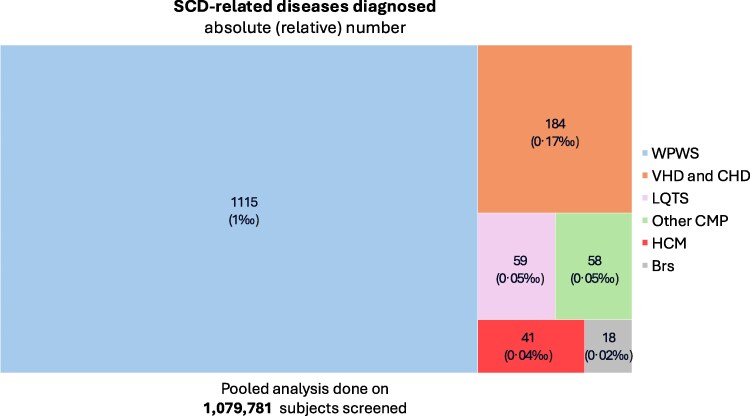
SCD-related diseases diagnosed during cardiovascular screening programmes. A pooled analysis was conducted on 1 079 781 screened subjects. The absolute (relative) number of conditions identified is here reported. BrS, Brugada syndrome (grey); CHD, congenital heart disease (orange); CMP, cardiomyopathies (green)—other CMP included dilated cardiomyopathy, arrhythmogenic right ventricular cardiomyopathy, left ventricular non-compaction and CMP not specified; HCM, hypertrophic cardiomyopathy (red); LQTS, long QT syndrome (pink); VHD, valve heart disease (orange). WPWS, Wolf-Parkinson-White syndrome (light blue).

The specific SCD-risk conditions are summarized in *Table 2* and *Figure 3*. WPW syndrome was the most frequently identified condition, with 1115 cases reported, corresponding to a pooled prevalence of 1‰. LQTS was the most frequently reported channelopathy, with 59 cases and a pooled prevalence of 0.05‰. Among cardiomyopathies, HCM was the most commonly diagnosed, with 41 cases and a pooled prevalence of 0.04‰.

**Table 2:** Cardiac disease detected during screening campaigns

First author	Year	N. of total participants	Cardiac condition detected	Cardiac disease at risk of SCD	CMP	CHAN	WPWS	Other cardiac disease at risk of SCD	Therapeutic interventions	SCD
R.W.A. Jones	1979	1034	1 (1‰)	1 (1‰)	··	··	1	··	1 digoxin	1 (normal ECG and autopsy)
N. Haneda	1986	128 454	78 (0.6‰)	41 (0.3‰)	1 HCM	··	··	33 CHD 3 AVB 4 VT/IVR	18 CHD corrections	··
H. Tasaki	1987	39 338	1025 (26‰)	213 (5.4‰)	··	··	38	3 AVB 172 CHD	··	··
K. Niwa	2004	152 322	202 (1.3‰)	202 (1.3‰)	··	··	198	1 VT 3 AVB	··	··
Y. Tanaka	2006	68 503	9 (0.1‰)	9 (0.1‰)	5 HCM 1 DCM	1 LQTS	1	1 PPH	··	3 (1 HCM, 2 low-risk no autopsy)
M.G. Wilson	2008	2720	9 (3.3‰)	9 (3.3‰)	1 ARVC	3 LQTS	4	1 VT	··	··
R. Providencia	2010	1472	··	··	··	··	.	··	··	··
J. Marek	2011	32 561	42 (1.3‰)	42 (1.3‰)	··	··	42	··	··	··
J. Fudge	2014	1339	52 (39‰)	5 (4‰)	··	··	5	··	··	··
N. Chandra	2014	11 845	16 (1.4‰)	16 (1.4‰)	16^a^	··	.	··	··	··
C. De Lazzari	2017	13 016	26 (2‰)	26 (2‰)	··	··	26	··	··	··
S.R. Nasr	2019	1412	2 (1.4‰)	2 (1.4‰)	1 ARVC	··	1	··	1 ablation	··
H.W. Liu	2020	566 447	14 517 (130‰)	682 (1.2‰)	44^a^ (15 HCM 3 DCM)	40 LQTS	523	29 Marfan 17 CHD 6 PPH 9 VT 13 AVB 1 VF	··	··
P. Vilardell	2020	1911	5 (2.6‰)	3 (1.6‰)	··	··	1	1 MVP 1 CCAA	1 ablation	··
H. Dhutia	2021	26 900	146 (5‰)	146 (5‰)^b^	14 HCM 3 ARVC 3 DCM 2 LVNC	10 LQTS 8 BRS 2CPVT	42	3 Marfan 58 CHD 1 AVB	22 drugs 3 ICD 26 ablations	··
A. Austin	2022	14 846	86 (5.7‰)	28 (1.8‰)	3 HCM	··	··	25 CHD	··	··
M Mancone	2022	11 949	25 (2.1‰)	25 (2.1‰)	2 HCM 2 DCM	4 LQTS 3 BRS	12	2 MIOC	··	··
M Abela	2023	2708	15 (5.5‰)	12 (4.4‰)	1 HCM	1 LQTS	5	2 CCAA 1 CHD 2 MVP	1 ablation 1 beta blocker	··
HK Lîm	2023	1004	62 (61.8‰)	43 (42.8‰)	··	··	4	23 MVP 16 CHD	4 ablations	··

AVB, atrioventricular block type III or advanced; CMP, cardiomyopathies; CHAN, channelopathies; WPWS, Wolff-Parkinson-White syndrome; SCD, sudden cardiac death; HCM, hypertrophic cardiomyopathy; DCM, dilated cardiomyopathy; ARVC, arrhythmogenic right cardiomyopathy; LVNC, left ventricular non-compaction; LQTS, Long QT syndrome; BRS, Brugada syndrome; CPVT, catecholaminergic polymorphic ventricular tachycardia; CCAA, congenital artery anomaly; CHD, congenital heart disease; MIOC, myocarditis; PPH, primary pulmonary hypertension; ICD, implantable cardioverter-defibrillator; VT, ventricular tachycardia; VF, ventricular fibrillation; IVR, idioventricular rhythm.

^a^The type of cardiomyopathy (CMP) diagnosed was not specified.

^b^Congenital heart diseases (CHD) were originally excluded by the authors in the manuscripts as SCD-risk conditions, obtaining a number of SCD-risk conditions of 88 (0.32%).

### Meta-analysis results: prevalence of cardiac conditions at risk of juvenile sudden cardiac death

For the prevalence of detected cardiac conditions, eighteen studies, totalling 1 078 309 participants, were included in the proportion meta-analysis of detected cardiac conditions. The pooled prevalence, estimated using a random-effects model, was 2.23 events per 1000 participants (95% CI: 0.94–5.29). Extremely high heterogeneity was observed among the studies (*I*^2^ = 100%, *P* < 0.0001), as illustrated in Supplementary material online, *Figure S1*. Subgroup analysis by screening modality showed a prevalence of 1.33 per 1000 (95% CI: 1.18–1.51; *I*^2^ = 0%) in studies using only ECG (*n* = 5) and 2.70 per 1000 (95% CI: 0.86–8.49; *I*^2^ = 100%) in those using a questionnaire plus ECG (*n* = 13). However, the difference between subgroups was not statistically significant (*P* = 0.23). Stratification by risk of bias also showed no significant differences between subgroups (*P* = 0.28). These stratifications are illustrated in Supplementary material online, *Figures S2* and *S3*.

**Figure 4:**
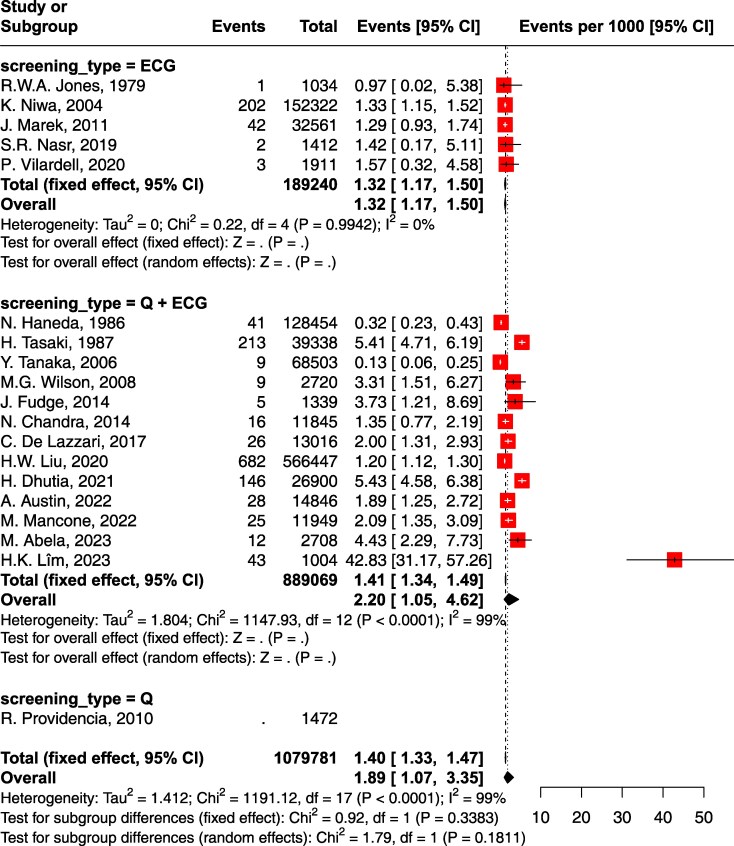
Stratified meta-analyses, based on type of screening, regarding conditions at risk for sudden cardiac death, per 1000 participants.

For the prevalence of cardiac diseases at risk of SCD, eighteen studies, totalling 1 078 309 participants, were included in the proportion meta-analysis of detected cardiac conditions. The pooled prevalence was 1.89 events per 1000 participants (95% CI: 1.07–3.35), with very high heterogeneity (*I*^2^ = 99%, *P* < 0.0001), as shown in *Figure 4*. The subgroup analysis revealed a prevalence of 1.32 per 1000 (95% CI: 1.17–1.50; *I*^2^ = 0%) in studies with ECG only (*n* = 5) and 2.20 per 1000 (95% CI: 1.05–4.62; *I*^2^ = 99%) in those with a questionnaire plus ECG (*n* = 13), with no statistically significant difference between the two groups (*P* = 0.18). Similarly, no significant differences emerged between subgroups based on risk of bias (*P* = 0.25). All these stratifications are illustrated in Supplementary material online, *Figures S4* and *S5*.

### Meta-analysis results: sensitivity analyses

The leave-one-out influence analysis (see Supplementary material online, *Figures S6* and *S7*) confirmed the robustness of our findings, showing that removing any single study did not substantially alter the pooled estimates. For example, regarding the rate of detected cardiac conditions (see Supplementary material online, *Figure S7*), the overall summary pooled rate per 1000 participants (Random) was 2.3 (95% CI 1, 5.4). When the neonatal study^38^ was omitted, the pooled rate remained similar at 2.4 (95% CI 1, 5.9).

In a pre-specified sensitivity analysis excluding studies with an event rate higher than 7 per 1000 participants, the results changed. For detected cardiac conditions (*n* = 15 studies, Supplementary material online, *Figure S8*), the pooled prevalence decreased to 1.23 per 1/000 (95% CI: 0.59–2.56), with heterogeneity remaining high (*I*^2^ = 98%). For diseases at risk of SCD (*n* = 17 studies, Supplementary material online, *Figure S9*), the pooled prevalence decreased to 1.57 per 1000 (95% CI: 0.99–2.50), with heterogeneity still at 98%.

### Patient management, follow-up and cost-effectiveness

Regarding patient management, in the study by Dhutia *et al*., 56% of individuals considered at-risk of juvenile SCD required further interventions during follow-up, including pharmacotherapy (25%), device implantation (3%), and transcatheter ablation of cardiac arrhythmias (30%). Abela *et al*. reported ablation for WPWS and beta-blocker therapy for LQTS, also emphasizing lifestyle modifications and family cascade screening. Nasr *et al*. reported ablation for WPWS and extensive follow-up for suspected ACM. Lîm *et al*. reported successful transcatheter ablation in four WPWS cases.

Seven studies included follow-up information, with only two studies reporting cases of juvenile SCD (*Table 2*). Tasaki *et al*., in a 6-year longitudinal study, highlighted the dynamic nature of paediatric cardiac health, showing changes in diagnostic yield over time and underscoring the need for regular re-evaluations. Three cases of juvenile SCD occurred in the study by Tanaka *et al*.: an event was attributed to HCM, while two events occurred in subjects classified as healthy during the screening programme. Jones *et al*. reported one case of juvenile SCD in a 6-month-old child without prior ECG abnormalities, whose aetiology remained unclear.

Dhutia *et al*. assessed the costs associated with screening using a combination of a questionnaire and ECG. Although the cost of screening with ECG was higher, this approach proved more efficient, with a 36% reduction in cost per SCD-risk conditions identified compared with using the questionnaire alone. Tanaka *et al*. were the only researchers who performed a cost-effectiveness analysis. They calculated that the total number of life years gained from the screening programme was 176, resulting in an estimated cost of $8800 per year of life saved by the screening programme.

## Discussion

This systematic review provides the most comprehensive synthesis to date of cardiovascular mass screening for SCD in young individuals, a research priority highlighted by both European and American Guidelines.^11,12^ The most frequently identified diagnoses were WPW syndrome, HCM, and LQTS. In this mixed cohort of athletes and non-athletes, the pooled prevalence of SCD-risk conditions was 1.4 cases per 1000 individuals screened. To further investigate and quantify this finding, our meta-analysis of eighteen studies calculated a similar pooled prevalence of 1.89 cases per 1000 (95% CI: 1.07–3.35) for these specific at-risk conditions. A crucial finding from our analysis is the extremely high statistical heterogeneity across studies (*I*^2^ ≈ 99–100%), which explains the slight variations in estimates and highlights the different methods and reporting standards in the field.

To provide context for this finding, the diagnostic yield of 1.4‰ (1.89‰ in our meta-analysis) is slightly lower than the 2–5‰ yield typically reported in screening programmes focused exclusively on competitive athletes. Athlete screening typically shows higher diagnostic yields because it targets older adolescents and young adults, age groups in which disease expression is more pronounced, and exercise-triggered phenotypes are more likely to manifest. In contrast, mass screening includes broader, younger populations, resulting in a naturally lower yield despite the use of similar (but not identical) tools. Conducting screening at a similar age to competitive athlete evaluations could further improve efficiency, as many inherited cardiac diseases manifest during late adolescence. From a health economics perspective, the lower yield of mass screening highlights the need for rigorous cost-effectiveness analyses to determine whether the potential benefits justify the resources required, particularly in comparison with established athlete screening programmes.^46^ Family cascade prenatal and neonatal screening, and pre-participation evaluations for athletes can detect specific high-risk individuals earlier, potentially reducing the incremental yield of subsequent population screening. However, these programmes generally target limited subgroups and are inconsistently applied across healthcare systems. In this context, a carefully designed population-based strategy could enhance standardization and equity of access. It should be stressed that its incremental clinical value requires further validation in settings with established early screening frameworks.

The extreme statistical heterogeneity (*I*^2^ ≈ 100%) suggests that a single pooled prevalence estimate should be interpreted with caution. This high variability likely stems from substantial differences across studies in screening protocols (e.g. ECG-only vs. screening combining questionnaires and ECG), population demographics (broad age ranges, variable inclusion of athletes, ethnic diversity), and the rigour of downstream second- and third-line diagnostic evaluation. Additionally, population context likely contributed to heterogeneity in diagnostic yield. For instance, the higher prevalence of CHD reported by Lîm *et al*. might reflect a rural Taiwanese cohort with limited neonatal screening, underscoring how healthcare access and demographic factors influence cross-study comparability. Therefore, the primary value of our synthesis lies not in a single prevalence figure but in the formal quantification of this diversity, underscoring the urgent need for standardized screening methodologies and reporting.

The earliest documented programme, conducted in the UK in 1979, was pioneering in the field and uniquely focused on newborns,^38^ while the largest initiative to date was carried out in Taiwan, where over a 10-year period, 566 447 students were screened.^28^ Notably, this single study accounts for approximately half of the total population analysed, and most screened individuals (88%) came from Asian countries. Recognizing the potential influence of such large or high-yield studies, the sensitivity analysis excluding studies with event rates above 7 per 1000 yielded a more conservative pooled prevalence of 1.57 per 1000 for diseases at risk of SCD, though statistical heterogeneity remained high.

The optimal timing for cardiovascular screening in the young remains a matter of debate. Although direct comparisons are hampered by heterogeneous data reporting, the prevalence of conditions at risk of SCD seems lower in younger children. For example, the prevalence of 0.3‰ in children aged 6–15 years found by Haneda *et al*.^37^ was ten times lower than the pooled prevalence in the entire population (1.4‰) and considerably lower than the pooled prevalence for at-risk conditions found in our meta-analysis (1.89‰). This aligns with ESC guidelines, which highlighted that in younger age groups, only certain conditions are typically detectable, such as CHD, specific channelopathies (CPVT and LQTS), or ACM.^9^ Nonetheless, the sensitivity analysis again showed that no single study, including the neonatal cohort, exerted undue influence on the pooled results or high heterogeneity found.

Longitudinal reassessment seems essential due to the age-dependent penetrance and variable expression of many inherited cardiac conditions,^47^ underscoring the importance of periodic screening over time,^48^ as also highlighted by Tanaka *et al*.^35^ School-based screening programmes seem to be a scalable approach for early detection of conditions at risk of SCD, as well as for promoting healthy habits (avoidance of illicit drugs and energy drinks) and providing Basic Life Support (BLS) training. Notably, since 2005, Denmark’s mandatory BLS training in schools has raised bystander CPR rates from 20% in 2001 to 77% in 2018, significantly improving survival from out-of-hospital cardiac arrest.^49^

Studies reveal inconsistencies in screening modalities and second-level investigations. Standardized questionnaires can improve consistency by incorporating family history and symptoms, as well as reproducibility. Wilson *et al*.^41^ emphasized the importance of demographic data, including smoking status. However, incomplete or unreliable data on illicit drug use pose challenges to accurate evaluation. Additionally, ECG screening faces issues such as undetected cases and overdiagnosis, often due to sport-related ECG changes.^50,51^ Standardizing ECG interpretation, as done in athletes,^44^ could again enhance accuracy. The subgroup meta-analysis directly explored this, revealing a numerically higher detection rate for conditions at risk of SCD in programmes using both a questionnaire and ECG (2.20 per 1000) compared with those using ECG alone (1.32 per 1000). However, this difference did not reach statistical significance (*P* = 0.18), suggesting that while a combined approach may be intuitively more comprehensive, its superiority in detection rate is not conclusively supported by the current aggregated data. This finding highlights the need for further standardized research to determine the optimal screening modality. Artificial intelligence shows promise in refining ECG analysis and improving consistency, though its application remains in early development and requires digital ECG acquisition.^52^

Following the screening programme, follow-up should include comprehensive cardiological evaluations with second- and third-line tests. Several studies^39,40,42^ have shown how structured referral pathways lead to accurate diagnoses and appropriate interventions. However, variability remains high. Excessive referral rates can increase healthcare costs and induce anxiety in the subject and the family, while relying solely on first-line screening (questionnaires and/or ECG) may significantly reduce sensitivity. In the absence of large-scale prospective studies, adherence to national and international diagnostic algorithms remains the most reliable strategy.^9,11,53^

The healthcare impact of early screening lies in its ability to modify the natural history of conditions at risk of SCD. Follow-up studies indicate that many individuals diagnosed with the condition require interventions, including pharmacotherapy, catheter ablation, and device implantation. Dhutia *et al*. reported that more than half of the identified cases underwent further management. Similarly, Abela *et al*., Nasr *et al*., and Lîm *et al*. documented successful interventions to mitigate the arrhythmic risk. This may improve long-term outcomes.

However, a substantial heterogeneity in follow-up data underscores the need for structured, long-term monitoring to accurately capture the incidence of ventricular arrhythmias and SCD. This clinical variability is directly mirrored by the extreme statistical heterogeneity (*I*^2^ ≈ 99–100%) identified in our meta-analysis, reinforcing the challenge of drawing a single, unified conclusion from the current body of literature. Future research should either include randomized comparisons between screened and unscreened populations or establish regional or national registries to ease longitudinal follow-up, support large-scale epidemiological research, and encourage international collaboration through standardized data integration.

Finally, the cost-effectiveness of questionnaire-plus-ECG screening in asymptomatic youth remains largely underexplored. Only one study in this review suggested a favourable cost-benefit ratio for the combined approach.^35^ However, recent evidence suggests that cost-effectiveness is highly sensitive to the specific screening and management models applied. For instance, the choice of risk stratification thresholds and time horizons significantly impacts economic viability,^54^ while other analyses suggest that targeting specific subgroups with high healthcare utilization may represent a strategic opportunity for early detection.^55^ Although ECG increases upfront costs, it may reduce long-term expenses by preventing severe events and the need for advanced treatments. While Maron *et al*.^16^ and Leslie *et al*.^20^ noted challenges such as low prevalence, high false-positive rates, and concerns about cost and equity, recent advances, such as AI-enhanced ECG interpretation, may improve specificity, reduce costs, and mitigate the risk of misinterpretation by inexperienced cardiologists. Further research should assess long-term sustainability by comparing advanced tools (e.g. online questionnaires, self-ECG, Artificial Intelligence^56,57^ with mass BLS training (early diagnosis vs. early intervention).

Enhancing screening accuracy and improving patient management require a unified approach. Risk stratification should combine clinical history, ECG, imaging, and genetic data to guide follow-up and detect hereditary conditions. Cascade family screening helps identify at-risk relatives, yet few studies included genetic testing.^33,39^ Diagnostic efforts must be paired with follow-up strategies. Research should focus on refining screening and implementing practical, evidence-based interventions (i.e. avoiding known arrhythmic triggers^58^ alongside pharmacological and interventional treatments) to reduce juvenile SCD risk and ensure sustainability. Furthermore, a concern regarding large-scale screening is the potential for high false-positive rates, which can cause downstream costs and lead to psychological distress. While the aggregated data in this study did not allow a pooled statistical analysis of false-positive rates due to inconsistent reporting, the wide variation in referral rates for second-line investigations suggests substantial heterogeneity in the specificity of initial screening protocols. High false-positive rates have historically challenged the viability of mass screening^59^; however, the adoption of refined ECG interpretation criteria has been shown to significantly improve specificity.^43-45^ Future screening strategies should prioritize minimizing false positives to ensure the clinical and economic sustainability of such programmes.

Limitations include the potential to omit relevant studies despite rigorous search and adherence to PRISMA guidelines. Including only studies with ≥1000 participants may have excluded smaller but informative research. Variation across countries and healthcare systems may affect generalizability. A significant limitation of this systematic review was the inability to conduct age-stratified meta-analyses. This was not due to variability in inclusion criteria or age ranges across studies, but rather to the lack of disaggregated age-specific data within individual studies. Most primary studies provided a single, aggregated outcome for their entire sample's age range, rather than providing separate results for distinct age subgroups (e.g. adolescents vs. young adults). As the source data was already aggregated, subgroup analysis based on age could not be performed.

## Conclusions

This systematic review reinforces the potential of combined questionnaire- and ECG-based screening to identify young individuals at increased risk of sudden cardiac death. Yet, current evidence remains geographically and demographically limited, with major gaps in standardisation, long-term outcomes, ethnic representation, and cost-effectiveness. Addressing these gaps through large-scale, diverse, and methodologically robust studies is essential to refine screening strategies, evaluate their real-world impact, and guide the development of equitable and sustainable public health policies aimed at preventing sudden cardiac death in the young.

## Supplementary Material

qcag004_Supplementary_Data

## Data Availability

All extracted data are directly available within the manuscript. However, upon request to the corresponding author, the complete data extraction table will be sent to whomever has requested it.
