# Supplementary figures and images for: Population-based screening for conditions associated with juvenile sudden cardiac death: a systematic review and meta-analysis
Source: Eur Heart J Qual Care Clin Outcomes. 2026 Jan 28;12(4):528–41. doi: 10.1093/ehjqcco/qcag004 (PMC13288737; doi:10.1093/ehjqcco/qcag004)

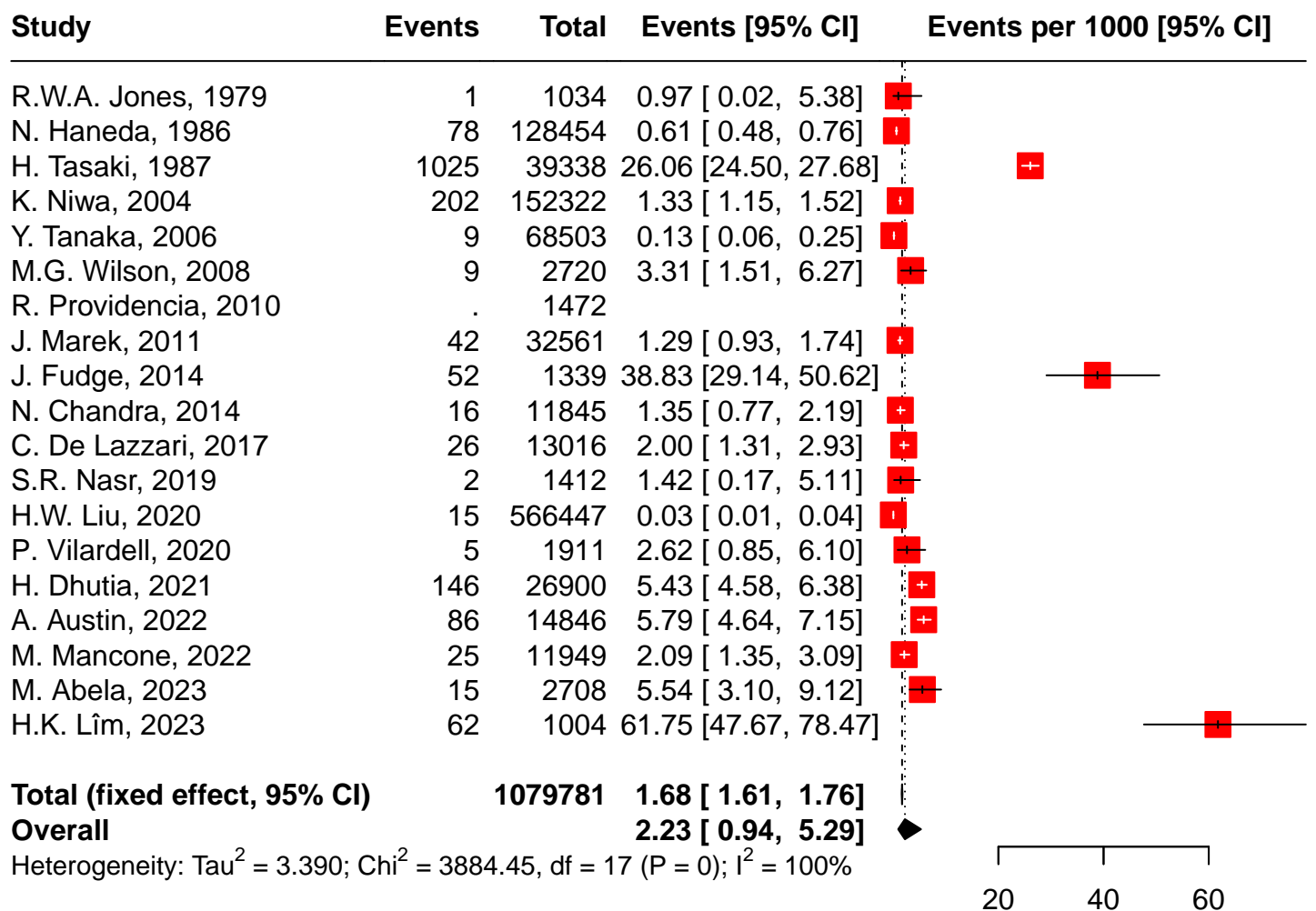

Supplement: qcag004_Supplementary_Data [file qcag004_supplementary_data.zip › Supplemental Figure 1.pdf]

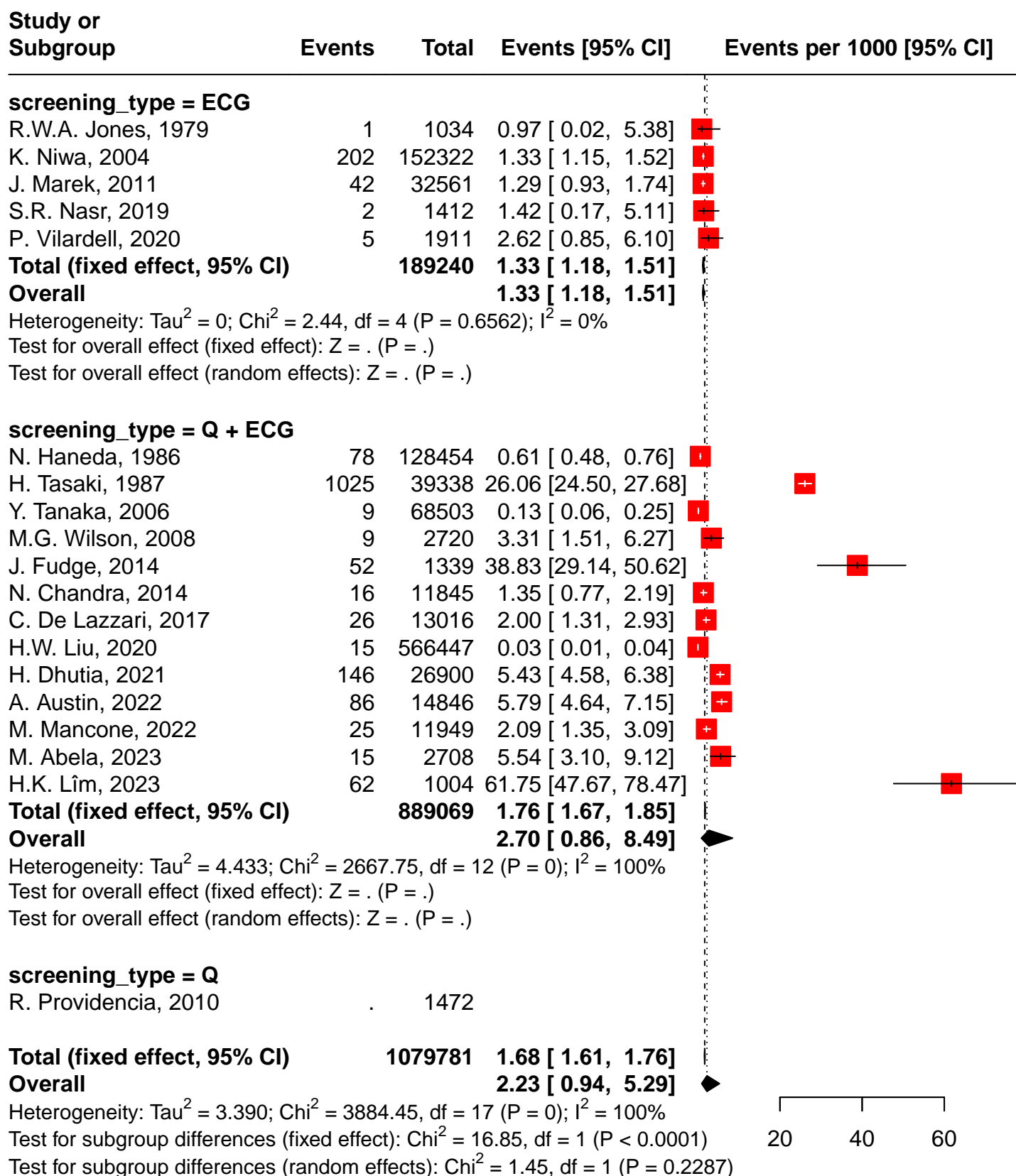

Supplement: qcag004_Supplementary_Data [file qcag004_supplementary_data.zip › Supplemental Figure 2.pdf]

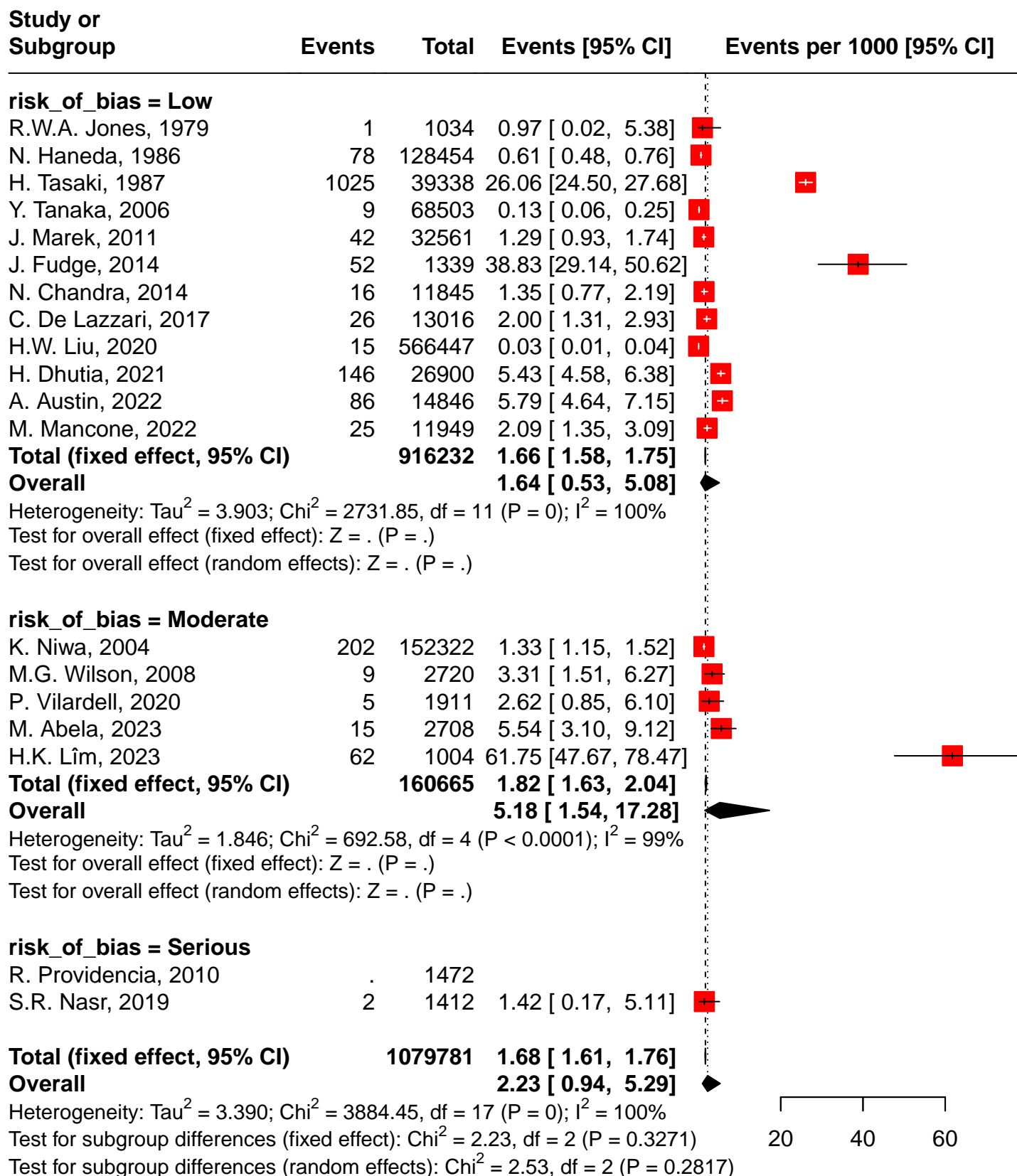

Supplement: qcag004_Supplementary_Data [file qcag004_supplementary_data.zip › Supplemental Figure 3.pdf]

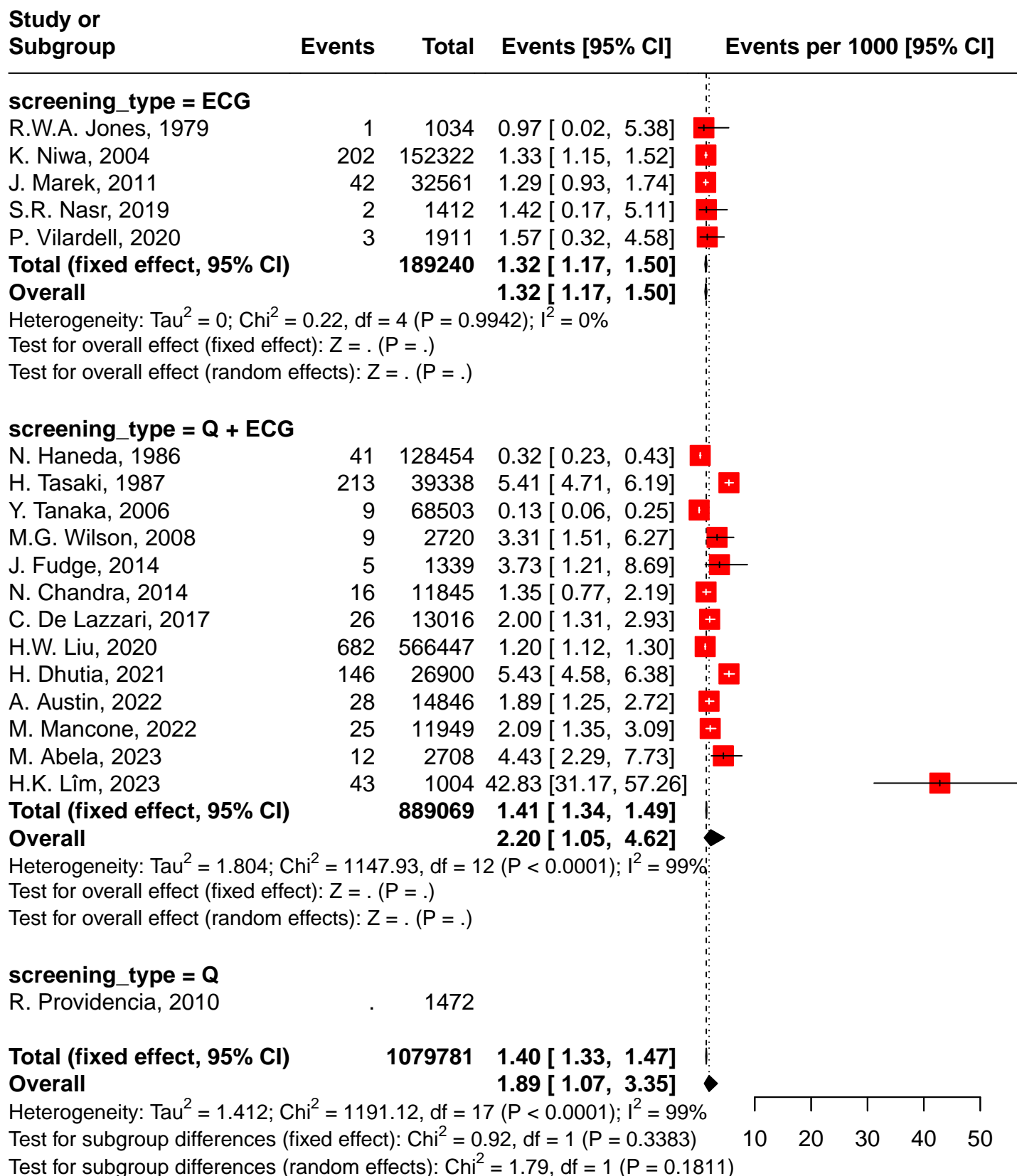

Supplement: qcag004_Supplementary_Data [file qcag004_supplementary_data.zip › Supplemental Figure 4.pdf]

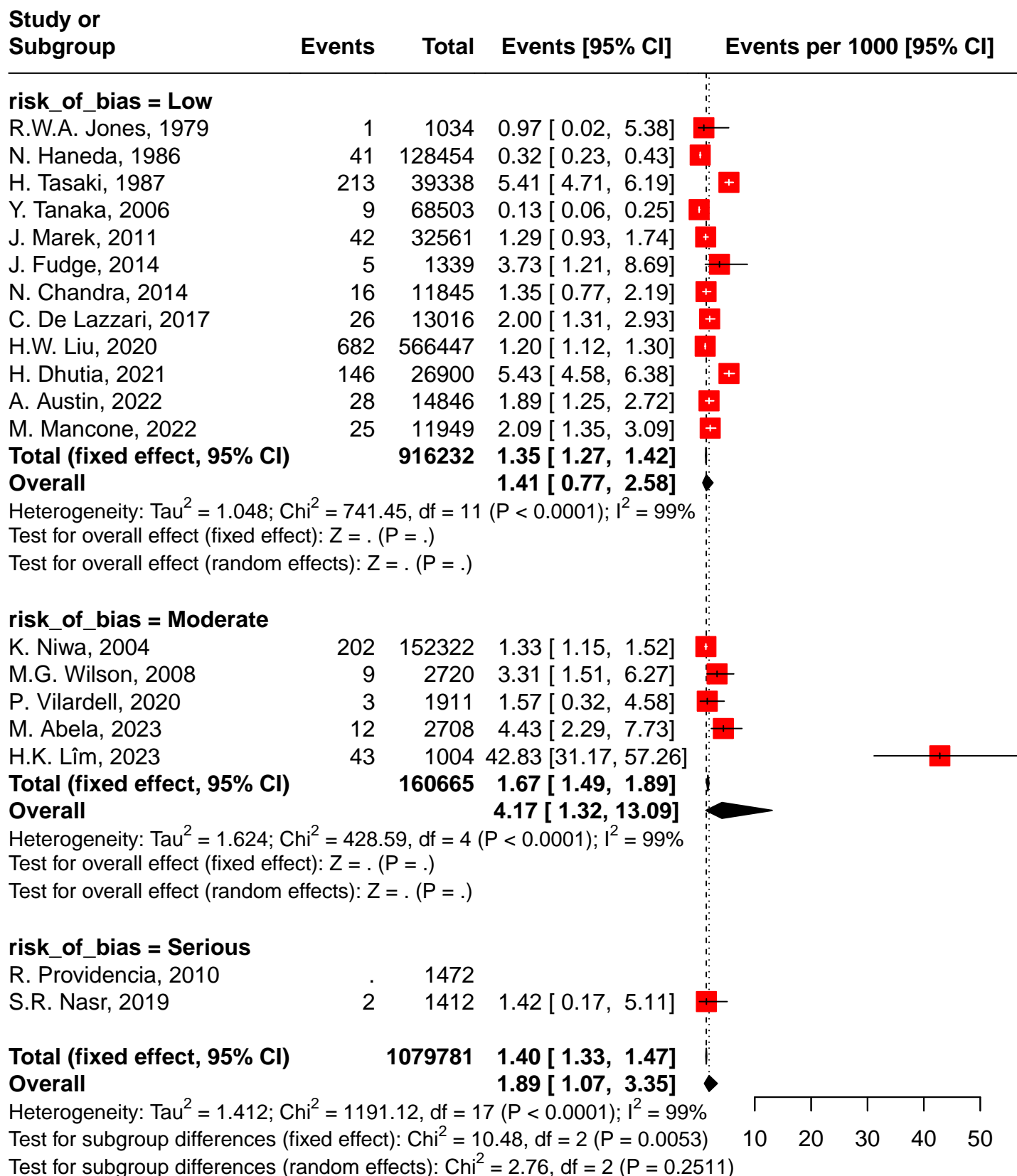

Supplement: qcag004_Supplementary_Data [file qcag004_supplementary_data.zip › Supplemental Figure 5.pdf]

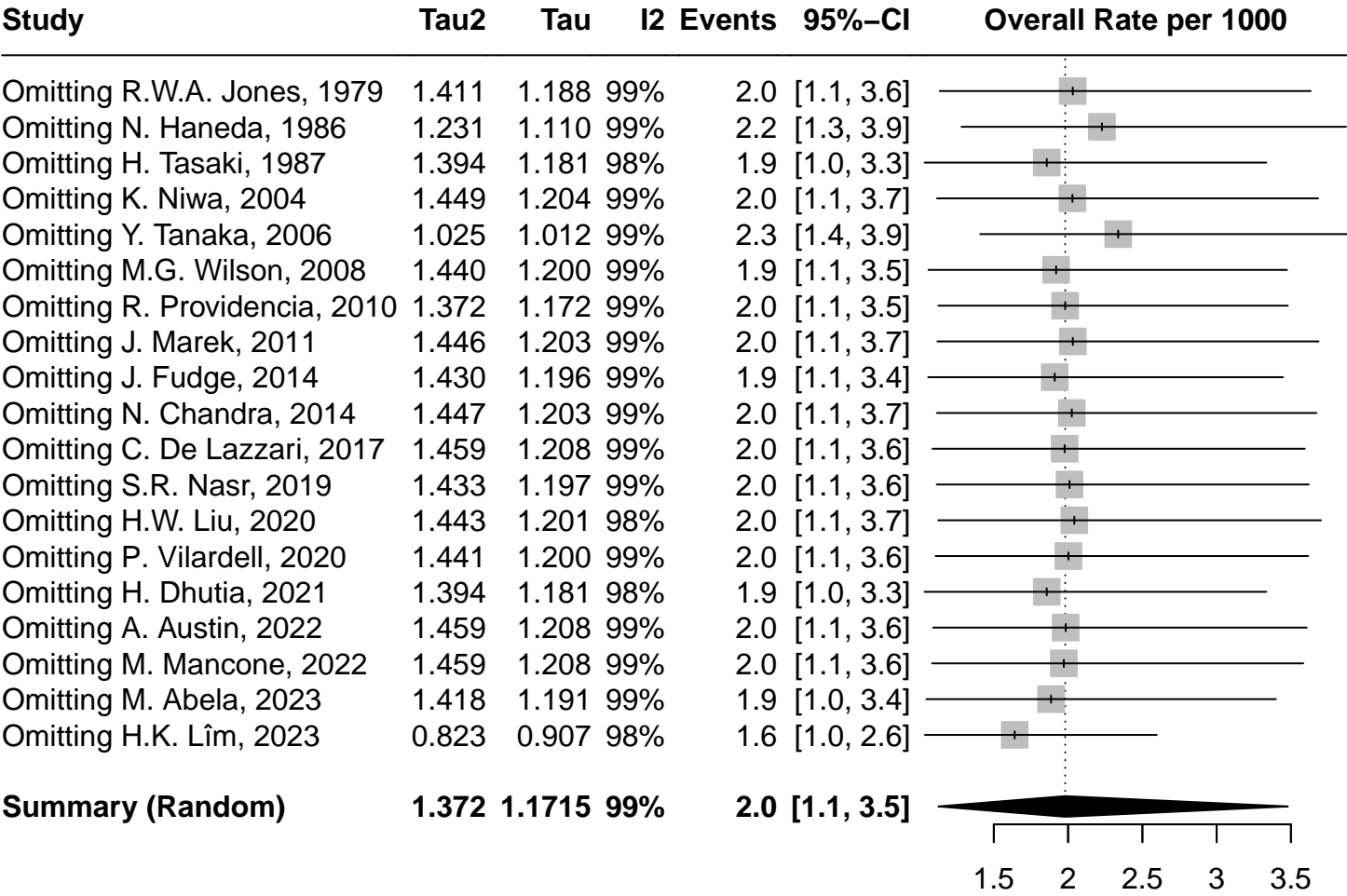

Supplement: qcag004_Supplementary_Data [file qcag004_supplementary_data.zip › Supplemental Figure 6.pdf]

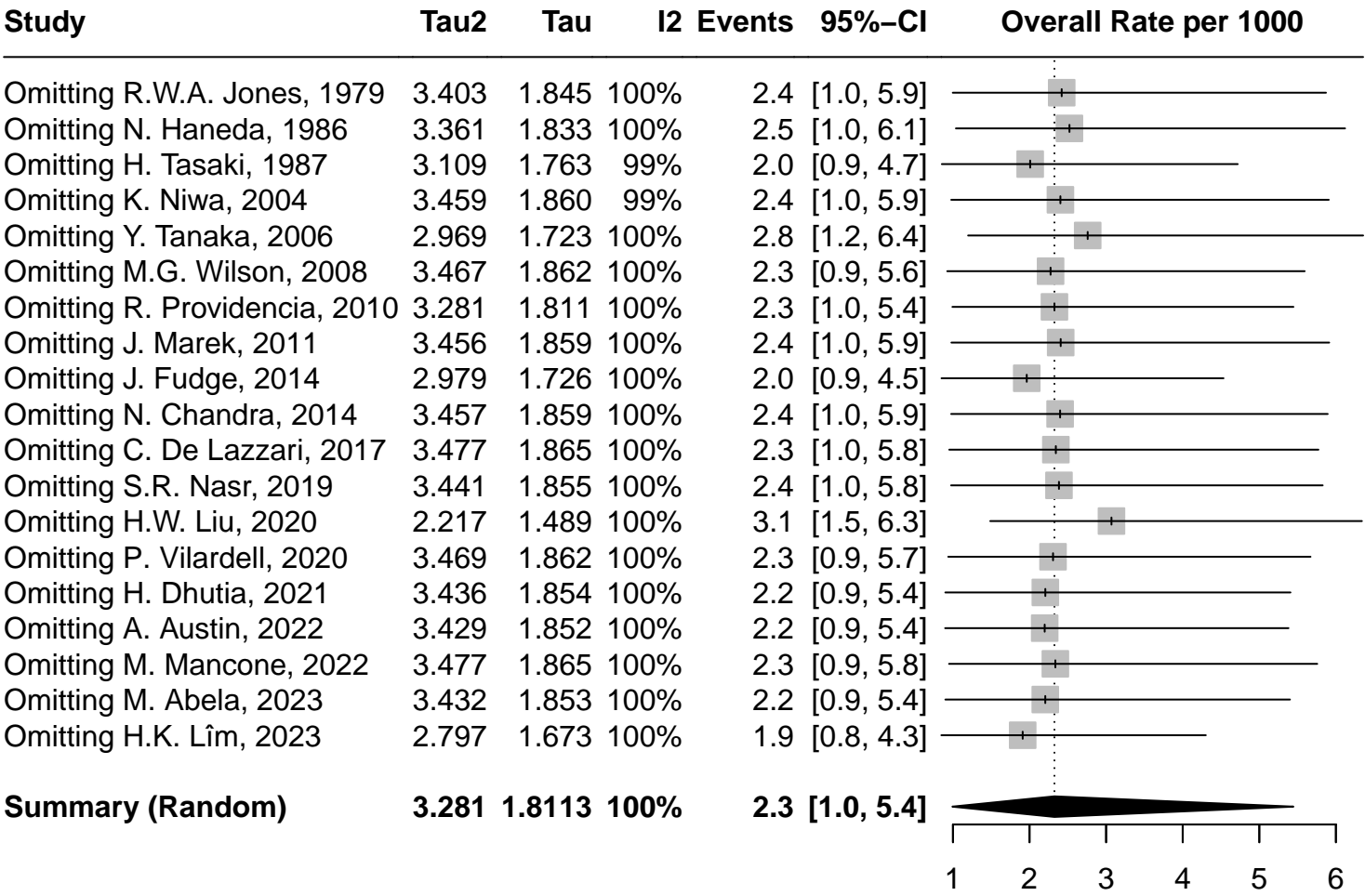

Supplement: qcag004_Supplementary_Data [file qcag004_supplementary_data.zip › Supplemental Figure 7.pdf]

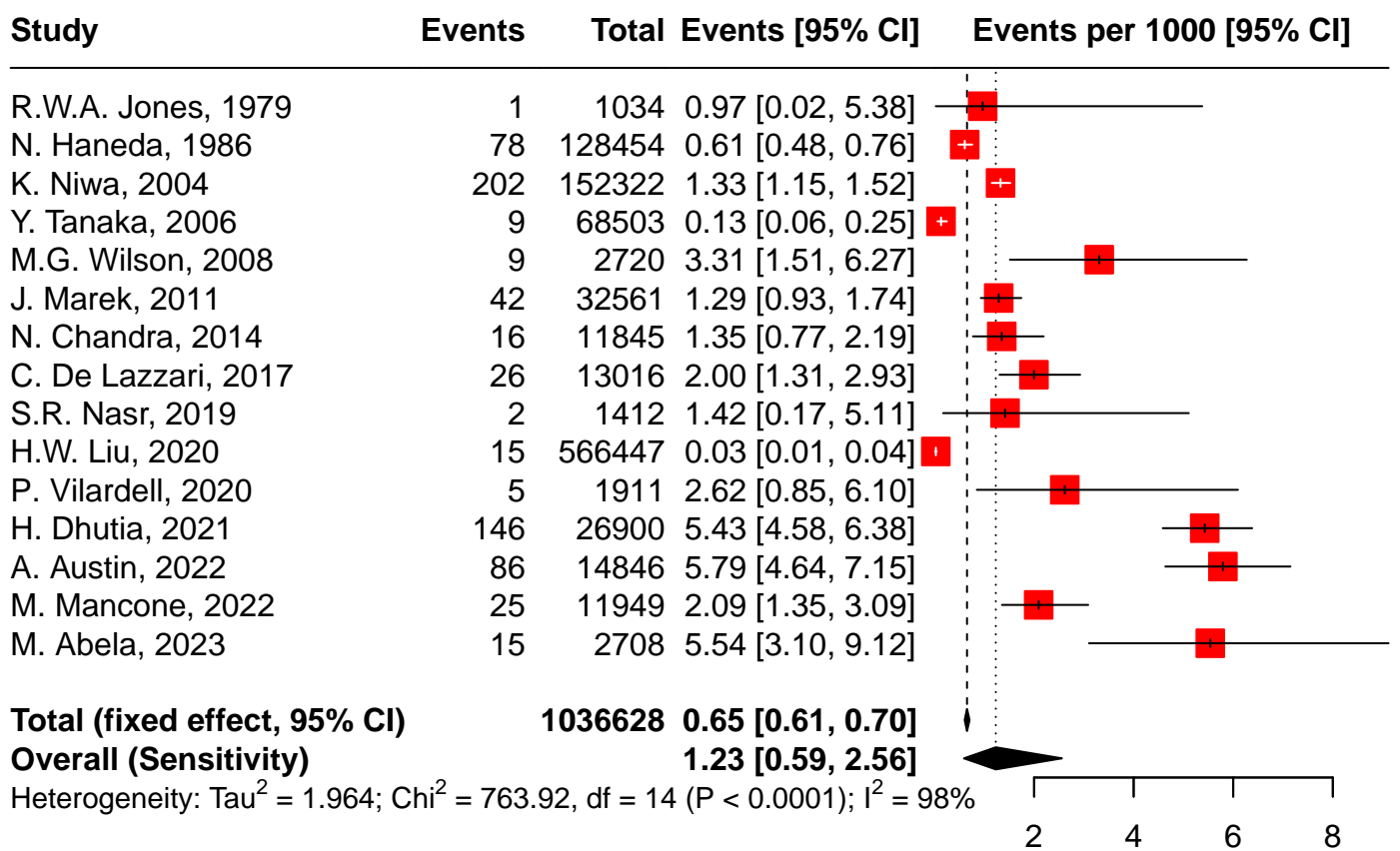

Supplement: qcag004_Supplementary_Data [file qcag004_supplementary_data.zip › Supplemental Figure 8.pdf]

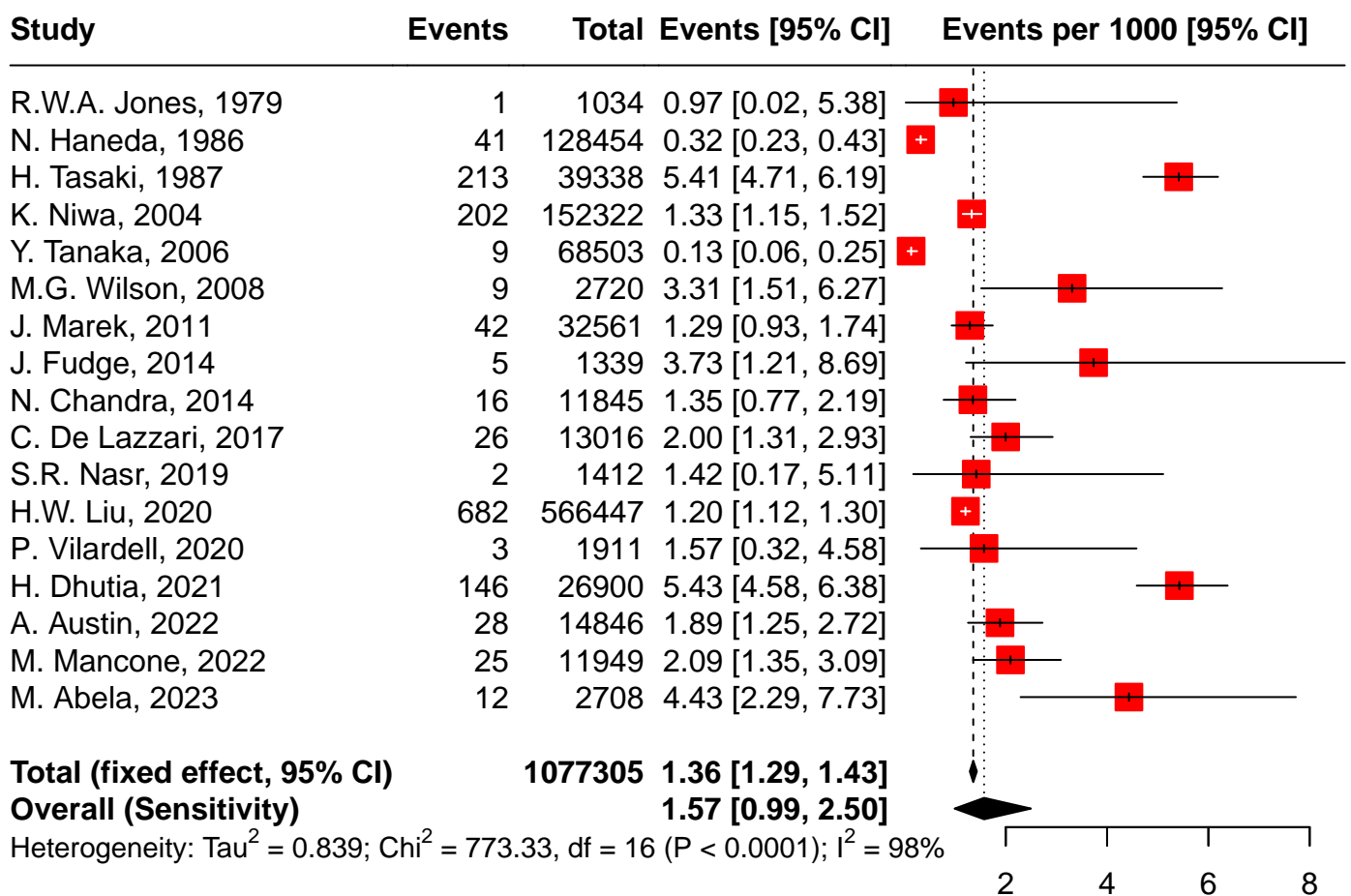

Supplement: qcag004_Supplementary_Data [file qcag004_supplementary_data.zip › Supplemental Figure 9.pdf]
